# Aging Suppresses Skin-Derived Circulating SDF1 to Promote Full-Thickness Tissue Regeneration

**DOI:** 10.1016/j.celrep.2018.08.054

**Published:** 2018-09-25

**Authors:** Mailyn A. Nishiguchi, Casey A. Spencer, Denis H. Leung, Thomas H. Leung

**Affiliations:** 1 Department of Dermatology, University of Pennsylvania School of Medicine, Philadelphia, PA 19104, USA; 2 Corporal Michael Crescenz Veterans Affairs Medical Center, Philadelphia, PA 19104, USA; 3 School of Economics, Singapore Management University, Singapore 188065, Singapore; 4 Institute for Regenerative Medicine, University of Pennsylvania, Philadelphia, PA 19104, USA; 5 These authors contributed equally; 6 Lead Contact

## Abstract

Physicians have observed that surgical wounds in the elderly heal with thinner scars than wounds in young patients. Understanding this phenomenon may reveal strategies for promoting scarless wound repair. We show that full-thickness skin wounds in aged but not young mice fully regenerate. Exposure of aged animals to blood from young mice by parabiosis counteracts this regenerative capacity. The secreted factor, stromal-derived factor 1 (SDF1), is expressed at higher levels in wounded skin of young mice. Genetic deletion of SDF1 in young skin enhanced tissue regeneration. In aged mice, enhancer of zeste homolog 2 (EZH2) and histone H3 lysine 27 trimethylation are recruited to the SDF1 promoter at higher levels, and pharmacologic inhibition of EZH2 restores SDF1 induction and prevents tissue regeneration. Similar age-dependent EZH2-mediated SDF1 suppression occurs in human skin. Our findings counter the current dogma that tissue function invariably declines with age and suggest new therapeutic strategies in regenerative medicine.

## In Brief

Nishiguchi et al. show that aging promotes full-thickness tissue regeneration in mouse skin. Aging modulates chromatin accessibility at the SDF1 gene, and genetic deletion of SDF1 in young skin enhances tissue regeneration. Human skin also exhibits age-dependent SDF1 suppression. They identify a rare example where aging improves tissue function.

## Graphical Abstract

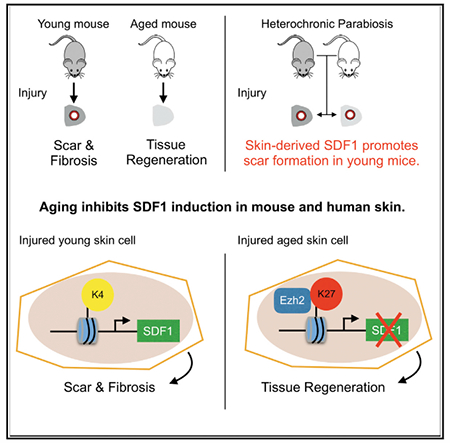

## INTRODUCTION

Organisms repair wounds using a combination of two biological processes: scar formation and tissue regeneration. Scar formation results in deposition of fibrous tissue that disrupts the original tissue architecture (Sun et al., 2014a). Tissue regeneration results in reconstitution of the original and functional tissue architecture, including all cellular subtypes and absence of scar formation (Carlson, 2007). Although amphibians regenerate lost limbs, mammals generally repair injured tissue with scar formation. However, limited examples of human tissue regeneration do exist, including adult liver regeneration, pediatric traumatic digit tip amputations, and fetal skin wounds. These examples suggest that the mechanisms mediating tissue regeneration remain conserved in mammals. Ear hole closure is a well-established and accepted animal model of mammalian tissue regeneration in multiple species, including rabbits (Goss and Grimes, 1975; Joseph and Dyson, 1966), the rodent strain *Acomys* (Seifert et al., 2012), and certain inbred and mutant mouse strains (Bedelbaeva et al., 2010; Clark et al., 1998; Leung et al., 2015; Shyh-Chang et al., 2013; Zhang et al., 2013). Repair of injured ear tissue exhibits similarities to amphibian limb regeneration, including basement membrane breakdown, blastema formation, and absence of scars (Brockes and Kumar, 2008; Clark et al., 1998; Gourevitch et al., 2003, 2009; Heber-Katz et al., 2013).

Human skin wounds invariably form scars. Aging slows the speed of skin re-epithelialization and the subsequent rate of wound repair (Gosain and DiPietro, 2004; Keyes et al., 2016), but the strength of re-epithelized skin remains roughly the same at any age (Kim et al., 2015). Dermatologists and plastic surgeons have observed that skin wounds in the elderly close with thinner scars. Indeed, the incidence of keloid and hypertrophic scar formation peaks in the second decade of life and decreases with age (Bayat et al., 2005; Sun et al., 2014b). These surprising and somewhat counterintuitive clinical observations suggest that the tissue-regenerative pathway in the skin, instead of being diminished, may be more effective in the elderly. Here we investigated the role of aging as a regulator of mammalian tissue regeneration.

## RESULTS

### Aging Promotes Tissue Regeneration and Decreased Scar Formation in Mouse Ears

**Figure 1.**
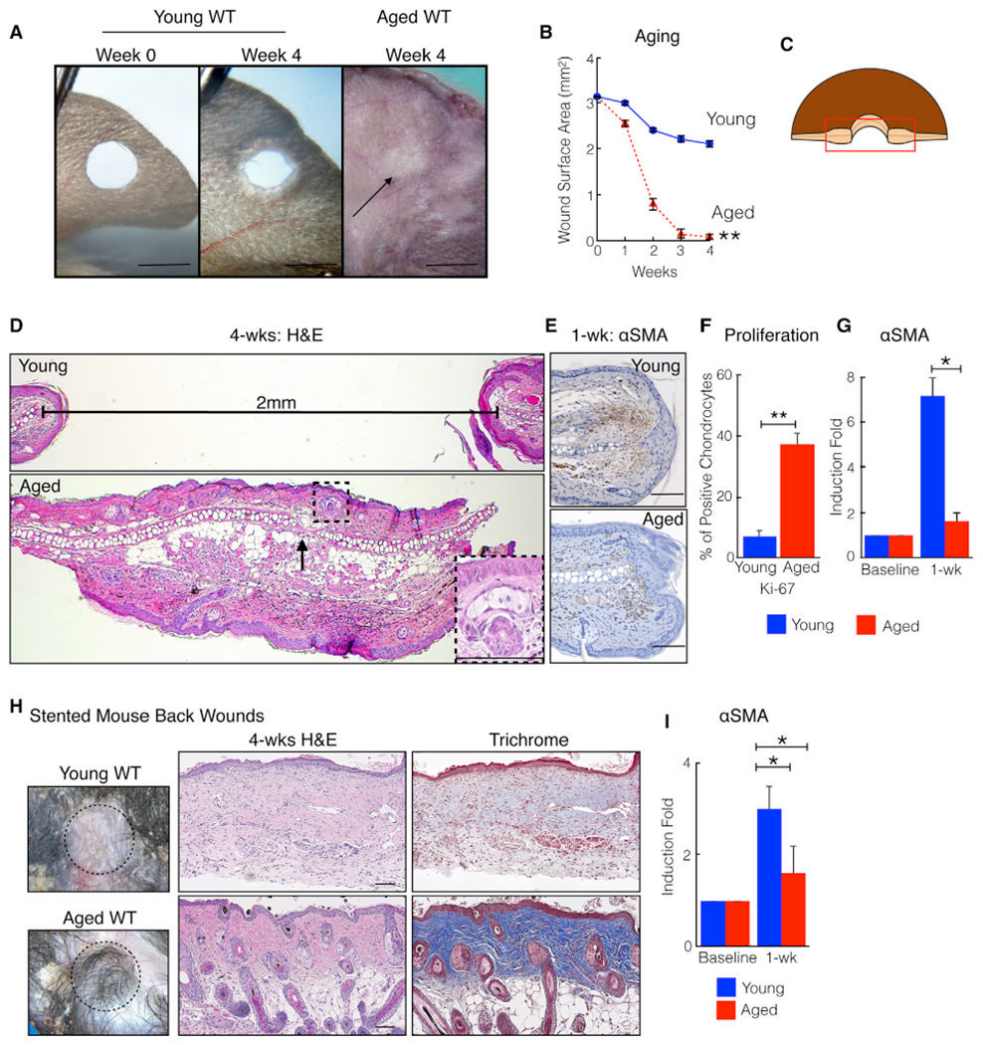
Aging Promotes Skin Tissue Regeneration in C57/BL6 Mice (A) Aged mice close ear holes to a smaller size. Shown are representative photographs of young WT and aged WT ears. The black arrow marks the healed hole. (B) Ear hole measurements. n = 15. **p = 0.003. (C) Schematic of histology orientation. (D) Aged mice heal ear holes with original tissue architecture, including cartilage, subcutaneous fat, and hair follicles: H&E staining. The horizontal line measures the distance between cartilage end plates. The box taken from the dotted square illustrates a regenerated hair follicle and sebaceous gland. (E) αSMA (brown cells) immunostaining of ears from young and aged mice. n = 10. (F) Ear tissue from injured aged mice demonstrates increased chondrocyte proliferation and decreased fibrosis. Shown are Ki-67-expressing chondrocytes 4 weeks post-injury. n = 4. **p = 0.002. (G) Relative αSMA transcript levels in ear wound edge tissue 1 week post-injury. n = 8. *p < 0.03. (H) Silicone-stented back wounds on aged mice exhibit improved tissue regeneration and diminished scar formation. Shown are representative photographs, H&E staining, and trichrome staining 4 weeks post-injury. Dark blue collagen represents normal skin; pale light blue collagen represents scar. n = 5. (I) Relative αSMA transcript levels in back wound edge tissue. n = 5. *p = 0.02. N, biological replicates per group. Error bars are SEM. Scale bars, 100 μM (histology) and 2 mm (photographs). See also Figure S1.

In young (1-month-old) wild-type (WT) mice, through-and-through 2-mm ear holes closed to a significantly larger size compared with aged (18-month-old) WT mice (Figures 1A and 1B). H&E staining of wound edge tissue from young mice revealed horizontally oriented fibroblasts and glassy thickened collagen, findings consistent with tissue fibrosis and scar formation (Figures 1C and 1D). Opposing cartilage end plates remained approximately 2 mm apart, confirming the absence of cartilage regeneration. By contrast, wound edge tissue from aged mice demonstrated normal tissue architecture, with hair follicles, sebaceous glands, and subcutaneous fat (Figure 1D, dotted box; Figure S1A). Significantly more chondrocytes expressed Ki-67, a cell proliferation marker (Figures 1F and S1B), and opposing cartilage end plates re-anastomosed (Figure 1D, black arrow). Compared with young mice, injured aged mice expressed significantly lower levels of α-smooth muscle actin (αSMA) mRNA and protein, a marker of myofibroblasts, which are involved in scar formation (Figures 1E and 1G). To assess other areas of skin, we performed full-thickness excisional wounding assays on dorsal back skin using silicone splints that minimize wound contraction and permit assessment of wound repair (Loh et al., 2009). In young mice, back wounds repaired with a fibrotic scar and exhibited increased levels of αSMA (Figures 1H, 1I, S1C, and S1D). By contrast, back wounds on aged mice repaired with diminished scar formation, as evidenced by return of hair follicles and lower levels of αSMA. Thus, age-dependent tissue regeneration may be generalized to mouse skin and may not require chondrocytes.

### A Circulating Factor Promotes Scar Formation

**Figure 2.**
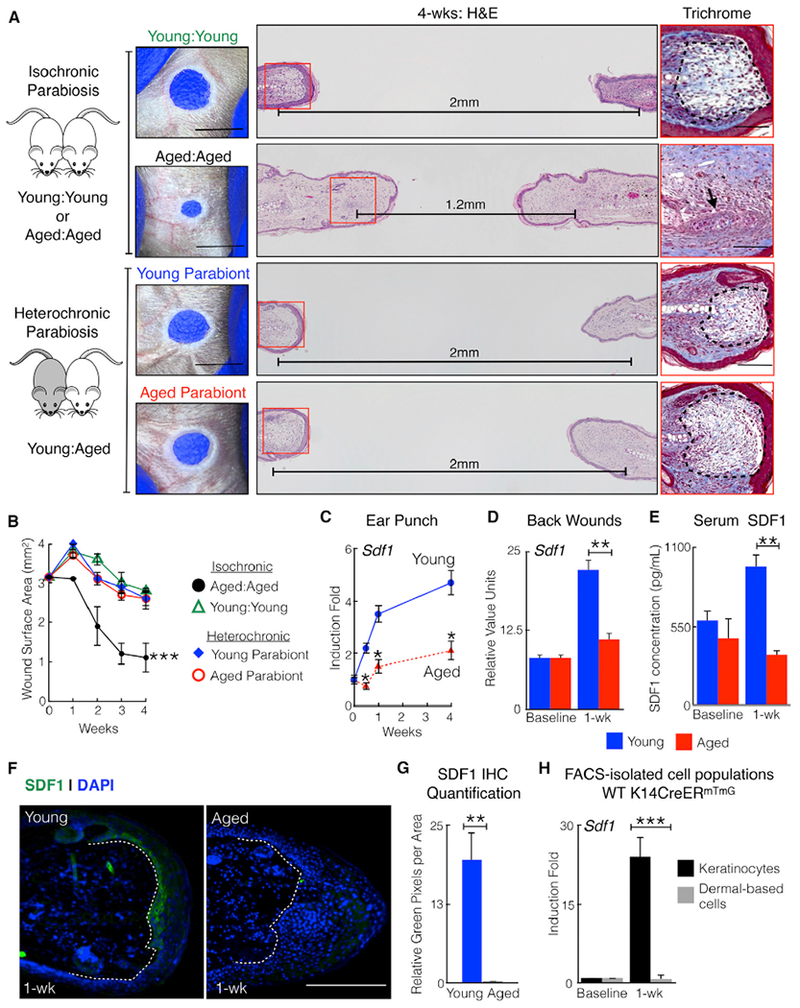
Injured Young Keratinocytes Secrete SDF1 to Promote Scar Formation (A) A circulating factor promotes scar formation in aged mice. Shown is a schematic of parabiosis pairs, photographs, H&E staining, and trichrome staining. The trichome images are taken from the red squares. Dotted areas mark scars. The black arrow marks chondrocyte proliferation. Horizontal lines indicate the distance between cartilage end plates. n = 5. (B) Ear hole measurements of individual parabionts. n = 5. ***p < 0.001, comparing aged: aged with young: young or either parabiont of young: aged. (C) Mice exhibit age-dependent SDF1 induction in ear and back wounds. Shown is the relative SDF1 transcript in ear wound edge tissue of young or aged WT mice. n = 24. *p < 0.03. (D) Relative SDF1 transcript in back skin wound edge tissue of young or aged WT mice. n = 6. **p = 0.004. (E) Ear injury induces SDF1 blood serum levels in young but not aged mice. Shown are SDF1 blood serum levels at baseline and 1 week after ear punch injury. n = 6. **p = 0.002. (F) Ear hole injury induces SDF1 expression in injured keratinocytes. Shown is SDF1 (green) immunostaining of ears from young and aged mice. Dotted lines identify the epidermal-dermal border. The hole is located to the right of the section. n = 6. (G) Quantification of SDF1 immunostaining. n = 6. **p < 0.01. (H) Relative SDF1 transcript in fluorescence-activated cell sorting (FACS)-isolated keratinocytes and dermally based cells in young mice. n = 6. ***p < 0.001. N, biological replicates per group. Error bars are SEM. Scale bars, 100 μM (histology) and 2 mm (photographs). Nuclei are counterstained blue. See also Figure S2.

In aged animals, exposure to young blood often improves stem cell function in many organs, including muscle, liver, spinal cord, and the brain (Brack et al., 2007; Conboy et al., 2005; Ruckh et al., 2012; Villeda et al., 2014). Given our age-defined phenotype, we assessed whether a circulating factor contributes to skin tissue regeneration. We generated heterochronic parabiosis pairs (Figures 2A and S2A), joining the vasculature of young and aged mice, and compared these with isochronic (young:young or aged:aged) control pairs. As expected, aged:aged pairs closed ear holes to a smaller size compared with young:young pairs (Figures 2A and 2B, black versus green lines). H&E staining of wound edge tissue from young:young pairs revealed scar formation, which is highlighted by Masson’s trichrome staining of collagen as a light blue region (Figure 2A, dotted areas; Figure S2B, low magnification). By contrast, wound edge tissue from aged:aged pairs demonstrated cartilage regeneration and the absence of scars (Figure 2A, black arrow). Within the heterochronic parabiosis pairs, the aged parabiont adopted the young parabiont fibrotic phenotype. Both parabiont mice repaired ear holes to a larger size and with scar formation (Figures 2A and 2B, red and blue lines). Thus, a circulating factor in young blood promotes scar formation and blocks skin tissue regeneration in aged mice. Compared with individual aged mice, the aged:aged pairs did not close their ear holes as effectively because the skin incision from the parabiosis procedure may elicit additional injury-induced circulatory factor(s). When we lengthened the time between the parabiosis procedure and ear injury, ear hole closure improved significantly (Figure S2C).

To identify potential circulating factors, we interrogated published RNA sequencing (RNA-seq) data generated from isolated keratinocytes from unwounded or wound edge tissue in young and aged C57/BL6 mice (Keyes et al., 2016). We focused on secreted factors and noted SDF1 (also known as *Cxcl12)* as one of their top hits. We previously demonstrated that SDF1-CXCR4 signaling modulated ear tissue regeneration in mice lacking *Cdkn1a* (Leung et al., 2015), and SDF1 was identified as a top regeneration-associated gene in a transcription profiling study comparing tissues from three regenerative animal models (King and Yin, 2016). Taken together, we postulated that suppression of SDF1 in aged mice promotes ear tissue regeneration. Compared with aged animals, ear punch or back skin injury in young animals significantly induced SDF1 mRNA and serum protein levels (Figures 2C–2E). Immunohistochemistry and cell isolation experiments localized the majority of SDF1 expression to wound edge keratinocytes (Figures 2F–2H and S2D). Thus, aging represses SDF1 secretion from injured keratinocytes.

### Injured Young Keratinocytes Secrete SDF1 to Promote Scar Formation

**Figure 3.**
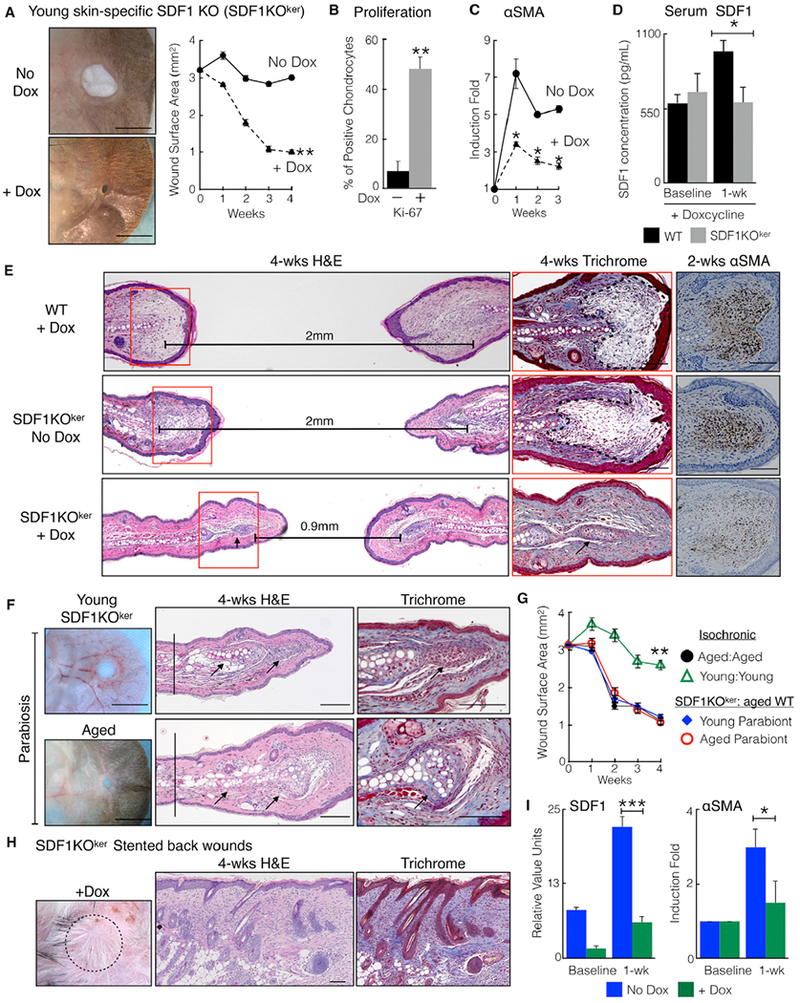
Skin-Specific SDF1 Knockout Mice Demonstrate Improved Tissue Regeneration (A) Young skin-specific SDF1 knockout mice (SDF1KO^ker^ mice) treated with doxycycline demonstrate improved ear hole closure. Shown are representative photographs and ear hole measurements. n = 6. **p = 0.007. (B) Ear wound edge tissue from doxycycline-treated SDF1 KO^ker^ mice exhibit increased chondrocyte proliferation and decreased fibrosis. Shown is quantification of Ki-67 immunostaining in chondrocytes 4 weeks post-injury. n = 6. **p < 0.01. (C) Relative αSMA transcript levels. n = 24. *p < 0.03. (D) Doxycycline-treated SDF1KO^ker^ mice do not induce SDF1 blood serum levels 1 week after injury. n = 6 for WT mice and n = 5 for SDF1 KO^ker^ mice. *p = 0.02. (E) Doxycycline-treated SDF1KO^ker^ mice regenerate injured ear tissue. Shown are H&E, trichrome, and αSMA staining (brown cells). Controls were doxycycline-treated wild-type (WT) mice and SDF1KO^ker^ mice without doxycycline. The trichome images are taken from the red squares. Dotted areas mark scars; the black arrow marks chondrocyte proliferation. Horizontal lines indicate the distance between cartilage end plates. n = 6. (F) Parabiosis between doxycycline-treated SDF1KO^ker^ mice and aged WT mice exhibiting improved ear hole closure. Shown are photographs of H&E staining, and trichrome staining. Black arrows indicate chondrocyte proliferation, and vertical lines indicate cartilage end plates. n = 3. (G) Ear hole measurements. n = 3. **p < 0.01, comparing young: young with either parabiont of young SDF1KO^ker^ :aged WT pair. (H) Stented back wounds on doxycycline-treated SDF1KO^ker^ mice demonstrate diminished scar formation. Shown are photographs of H&E staining, and tri chrome staining 4 weeks post-injury. n = 5. (I) Relative SDF1 and αSMA transcript levels in back skin wound edge tissue. n = 5. *p = 0.02, ***p = 0.0003. N, biological replicates per group. Error bars are SEM. Scale bars, 100 μM (histology) and 2 mm (photographs). Nuclei are counterstained blue. See also Figure S3.

To investigate whether the suppression of keratinocyte-secreted SDF1 promotes tissue regeneration, we generated *K5-rtTA; tetO-Cre; Cxcl12*^*fl/fl*^ mice (abbreviated to SDF1KO^ker^) and confirmed conditional SDF1 inactivation in keratinocytes (Greenbaum et al., 2013; Figure S3A). Compared with controls (doxycycline-treated WT mice or untreated SDF1KO^ker^ mice), doxycycline-treated SDF1KO^ker^ mice closed ear holes to a significantly smaller size (Figures 3A and S3B), and wound edge tissue revealed increased chondrocyte proliferation, diminished αSMA expression, and decreased scar formation (Figures 3B, 3C, 3E, S3C, and S3D). Moreover, injury did not increase serum SDF1 levels in SDF1KO^ker^ mice (Figure 3D). To confirm that skin-specific SDF1 promotes scar formation in heterochronic parabiosis, we generated parabiosis pairs between young doxycycline-treated SDF1KO^ker^ and aged WT mice. Both parabionts closed ear holes to a significantly smaller size, similar to the aged:aged pairs, and wound edge tissue from both parabionts demonstrated cartilage regeneration and decreased scar formation (Figures 3F and 3G, black arrows). Finally, compared with non-doxycycline-treated SDF1KO^ker^ mice, silicone-stented back wounds on doxycycline-treated SDF1KO^ker^ mice exhibited diminished scar formation, as evidenced by return of hair follicles and reduced levels of αSMA (Figures 3H, 3I, and S3E). Thus, circulating SDF1 in young blood originates from wounded keratinocytes to drive scar formation.

### Aging Suppresses SDF1 Activation via Increased Recruitment of EZH2 and H3K27me3 to the SDF1 Gene

**Figure 4.**
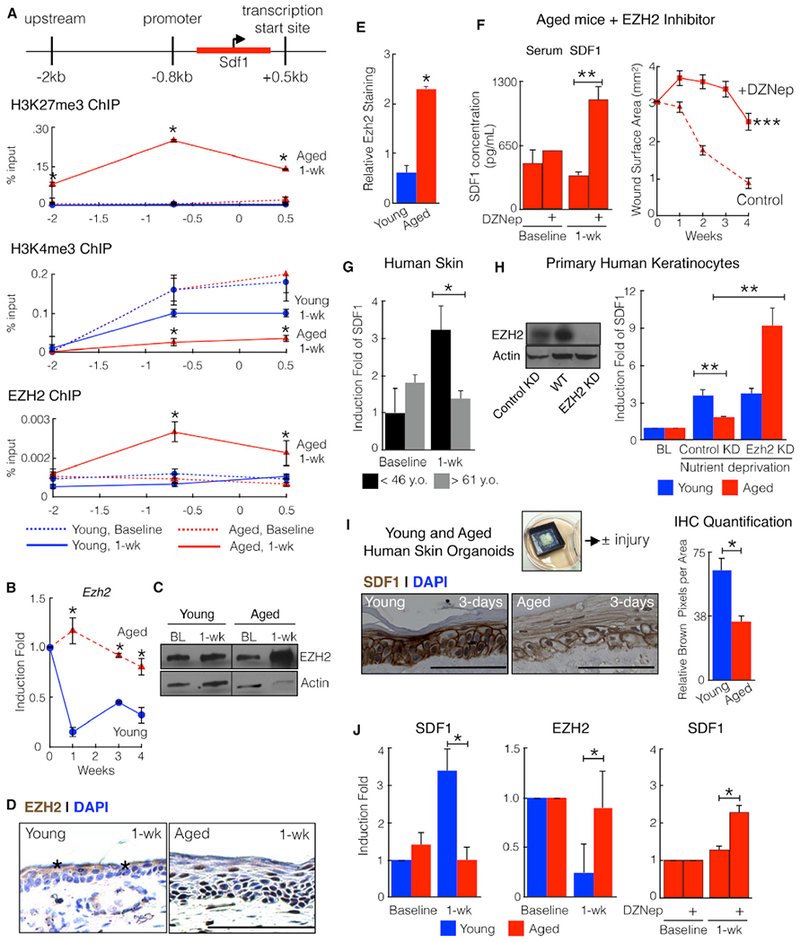
Mouse and Human Skin Exhibit Age-Dependent EZH2-Mediated SDF1 Induction (A) Age-dependent epigenetic regulation of SDF1. Shown are H3K27me3, H3K4me3, and EZH2 chromatin immune precipitation of ear wound edge tissue at baseline and 1 week post-injury at 3 different locations on the SDF1 gene. n = 6. *p < 0.05. (B–E) Injury-induced EZH2 increases with age. Shown are EZH2 transcript (B, n = 24), protein (C, n = 4), and immune staining (D, brown cells, black asterisks mark background staining, n = 4)(E) immunostaining quantification in ear wound edge tissue at baseline (BL) and 1 week post-injury. *p < 0.01. (F) Pharmacologic inhibition of EZH2 (DZNep) in aged mice rescues SDF1 induction and blocks tissue regeneration. Shown are SDF1 blood serum levels and ear hole measurements. n = 3. ***p = 0.00065. (G) Human skin exhibits age-dependent SDF1 induction. Shown are SDF1 transcript level sat baseline and 1 week post-injury in human skin of different ages. n = 5 patients < 46 years old (black bars) and n = 4 patients > 61 years old (gray bars). *p < 0.05. (H) EZH2 knockdown rescues SDF1 induction in aged human keratinocytes. Left: western blot analysis of EZH2 and actin in WT, control (scramble) knockdown (KD), and EZH2 knockdown keratinocytes. n = 4. Right: SDF1 transcript levels at baseline (BL) and after nutrient deprivation in WT, control knockdown, and EZH2 knockdown young and aged primary human keratinocytes. n = 4. **p < 0.01. (I) Young and aged human skin organoids exhibit age-dependent SDF1 induction. Shown are SDF1 immunostaining (brown cells) and quantification 3 days after hole punch. n = 3. *p < 0.05. (J) EZH2 inhibition rescues SDF1 induction in aged human skin organoids. Shown are SDF1 (left) and EZH2 (center) transcript levels in young (blue bars) and aged (red bars) human skin organoids and SDF1 transcript in aged human skin organoids with and without DZNep (right). n = 6. *p < 0.02. N, biological replicates per group. Error bars are SEM. Scale bars, 100 μM. Nuclei are counterstained blue. See also Figure S4.

How does aging modulate SDF1 expression? Known regulators of SDF1, *Cdkn1a* and *Cepba,* do not change with age (Leung et al., 2015; Figure S4A). Compared with young mice, tiled chromatin immunoprecipitation of wound edge tissue from injured aged mice exhibited increased enrichment for histone H3 lysine 27 trimethylation (H3K27me3), an epigenetic marker of gene inhibition, at the SDF1 promoter (−2 kb and −0.8 kb from the transcription start site) and transcription start site (TSS) (+0.5 kb from the transcription start site) (Figures 4A and S4B; Sen et al., 2016). The same tissue also demonstrated decreased histone H3 lysine 4 trimethylation (H3K4me3) enrichment, an epigenetic marker of gene activation. EZH2 catalyzes the addition of methyl groups to H3K27 (Sen et al., 2016). Compared with young mice, wound edge tissue from injured aged mice exhibited increased levels of EZH2 transcript and protein and increased EZH2 enrichment at the SDF1 promoter and transcription start site (Figures 4A–4E). Finally, aged mice treated with 3-Deazaneplanocin A hydrochloride (DZNep), a well-characterized pharmacologic inhibitor of EZH2, restored SDF1 induction, and ear holes closed with larger sizes (Figure 4F; Glazer et al., 1986). Taken together, aging suppresses SDF1 induction through increased recruitment of EZH2 to the SDF1 promoter.

### Human Skin Exhibits Age-Dependent EZH2-Mediated SDF1 Induction

To assess SDF1 expression in human skin, we interrogated published microarray data from uninjured and 1 week post-injured human skin of different ages (Nuutila et al., 2012; 2013; Stone et al., 2017). Similar to the mouse, wounded young human skin contained higher levels of SDF1 transcript compared with wounded aged human skin (Figure 4G). To elucidate the role of EZH2 in human skin, we knocked down EZH2 in human keratinocytes (Figure 4H). Nutrient deprivation, which mimics the loss of blood supply to tissue during injury and is a known stimulant of SDF1 (Santiago et al., 2011), significantly induced SDF1 in young but not aged keratinocytes, and EZH2 knockdown rescued SDF1 induction in aged keratinocytes (Figure 4H). To further test the relevance of our findings to human skin, we created three-dimensional human skin organoid constructs with young (<1 year old) and aged (>71 years old) human skin cells (Figures 4I, S4C, and S4D). Hole punch injury of these human skin equivalents induced SDF1 transcript and protein and down regulated EZH2 transcript in an age-dependent manner (Figures 4I and 4J). DZNep also restored SDF1 expression in aged organoids. Thus, our findings suggest that inhibition of SDF1 or EZH2 may be used to decrease scar formation in humans in potential future clinical trials.

## DISCUSSION

Our results counter the current dogma that tissue function inevitably worsens with age and uncovers potential mechanisms to explain the paradoxical effect of aging on skin tissue regeneration. Our ear tissue regeneration data agree with prior work on aging and speed of wound closure. Aging slows the speed of skin re-epithelialization on a dorsal back wound (Gosain and DiPietro, 2004; Keyes et al., 2016). In the ear, young animals reach ~85% of their final hole size by week 2 after injury, whereas aged animals attain ~30% of their final hole size. Thus, young ears repair faster but a scar develops, whereas aged ears repair slower but to a better resolution. Indeed, overexpression of SDF1 speeds up skin re-epithelialization (Vågesjö et al., 2018). We speculate that, from an evolution perspective, a young injured animal favors fast and imperfect wound repair over slow and perfect tissue regeneration. Future studies are needed to assess the relationship between skin re-epithelialization speed and scar formation.

This aging-induced switch between scar formation and tissue regeneration appears to be a gradual process rather than a binary decision. Prior students demonstrated that middle-aged (10-month-old) mice closed ear holes to a greater extent compared to 1-month old mice (Costa et al., 2009; Reines et al., 2009). Our results suggest that this ability further improves with age because we see full ear hole closure in 18-month-old mice (versus partial closure in 10-month-old mice shown in previous studies). We speculate that even older animals (> 18 months old) would behave similarly to 18-month-old animals but acknowledge the possibility that the tissue regeneration ability peaks at 18 months of age. Taken together, scar formation and tissue regeneration may indeed coexist and may not be mutually exclusive processes. More work is needed to understand how these two processes regulate each other. Finally, an alternative interpretation of our dataset is that regenerative healing is the “default” program and that young age inhibits this process. Although scar formation is the dominant form of wound repair in mammals at any age, we cannot rule out this possibility and include it in our discussion to be thorough.

Although our ear and back wound models are superficially similar, they still represent different systems with different cell types involved. We have found that keratinocyte-secreted SDF1 regulates the choice between tissue regeneration and scar formation in the ear and back skin. Increased SDF1 also drives scar formation in other organs, including mouse lung and zebrafish fin, and loss-of-function CXCR4 mutations, the canonical receptor for SDF1, promote liver regeneration (Ding et al., 2014a; Makino et al., 2013; Shu et al., 2013). Thus, our data support the attractive hypothesis that SDF1 regulates tissue regeneration across multiple organs, and future studies are needed to elucidate whether the precise cellular and molecular mechanisms are conserved in other organs.

Our parabiosis experiments suggest that injury-induced changes in serum SDF1 blood levels are due to skin-specific secretion. However, SDF1 signaling may propagate through autocrine feedforward loops (Kojima et al., 2010; Sauvé et al., 2009). Although our data suggest that skin-secreted SDF1 initiates the process, it remains possible that skin-secreted SDF1 activates or mobilizes circulating cells to produce additional SDF1. Finally, although skin-specific loss of SDF1 significantly improves skin tissue regeneration, knockout mice do not fully close injured ear holes. This suggests that other factors also likely participate in tissue regeneration.

Current treatment paradigms for hypertrophic and keloid scars are woefully ineffective and represent a major unmet need in clinical practice. Human pathologic scars contain higher amounts of SDF1, and keloid-prone individuals have higher circulating levels of SDF1 (Ding and Tredget, 2015). Human skin grafts transplanted onto the back of immune-deficient mice invariably form scars, and pharmacologic inhibition of SDF1 signaling reduced scar formation (Ding et al., 2014b). The discovery of a conserved pathway controlling age-dependent tissue regeneration suggests that clinical trials testing SDF1 and/or EZH2 antagonists in the treatment of pathological scarring in humans are warranted.

## STAR★METHODS

### KEY RESOURCES TABLE

**Table:** 

REAGENT or RESOURCE	SOURCE	IDENTIFIER
Antibodies		
Mouse monoclonal anti-Ki-67	Abcam	Cat#ab15580; RRID:AB_2088164
Rabbit polyclonal anti-SDF1	Abcam	Cat#ab25117; RRID:AB_2088164
Rabbit monoclonal anti-Ezh2 XP (clone D2CP)	Cell Signaling Technology	Cat#5246S; RRID:AB_10694683
Rabbit polyclonal anti-αSMA (clone 1A4)	Abcam	Cat#Ab7817; RRID:AB_262054
Rabbit polyclonal anti-Keratin 14 (clone Poly19053)	BioLegend	Cat#905304; RRID:AB_2616896
Rabbit polyclonal anti-Keratin 10 (clone Poly19054)	BioLegend	Cat#905404; RRID:AB_2616955
Rabbit polyclonal anti-Loricrin (clone Poly19051)	BioLegend	Cat#905104; RRID:AB_2616895
Rabbit polyclonal anti-β-actin-HRP (C-11)	Santa Cruz Biotechnology	Cat#sc-1615; Lot#G2313, A3105; RRID:AB_630835
Donkey Anti-Rabbit IgG H&L (Alexa Fluor 488)	Abcam	Cat#ab150073; RRID:AB_2636877
Rabbit monoclonal anti-Tri-Methyl-Histone H3 (Lys27) (clone C36B11)	Cell Signaling Technology	Cat#9733; RRID:AB_2616029
Rabbit polyclonal anti-trimethyl-Histone H3 (Lys4)	Millipore	Cat#07473; RRID:AB_1977252
ChIPAb+ EZH2 – ChIP Validated Antibody and Primer Set (Clone AC22)	Millipore	Cat#17-662; RRID:AB_1977568
Biological Samples		
Normal Adult Primary Human Keratinocytes	Penn Skin Biology and Diseases Resources-based Center (SBDRC)	https://dermatology.upenn.edu/sbdrc/
Normal Human Primary Keratinocytes	Penn SBDRC	https://dermatology.upenn.edu/sbdrc/
Microarray data for baseline and wounded human skin	Stone et al., 2017	GEO: GSE84571
Microarray data for baseline and wounded human skin	Nuutila et al., 2012	GEO: GSE28914
Microarray data for baseline and wounded human skin	Nuutila et al., 2013	GEO: GSE33169
Chemicals, Peptides, and Recombinant Proteins		
Meloxicam Solution for Injection (5mg/mL)	Putney	NDC: 26637-621-01; Lot#GI60154
Buprenorphine Hydrochloride, Injection (0.3mg/mL)	Butler Schein Pharmaceuticals	NDC: 42023-179-05; Lot#809762
TRI Reagent	Zymo Research	Cat#R2050-1-200; Lot#ZRC184119; ZRC182093
Lysing Matrix D (2mL Tube)	MP Biomedicals	Cat#116913050; Lot#104291
Maxima Reverse Transcriptase	Thermo Scientific	Cat#EP0741
Histone Methyltransferase EZH2 Inhibitor, DZNep	Millipore	Cat#252790; Lot#2704823; Lot#D00157396
Maxima Reverse Transcriptase	Thermo Scientific	Cat#EP0741
Doxycycline Grain-Based Rodent Diet (6g/kg)	Bio-Serv	Cat#14-727-450
Tamoxifen	Sigma	Cat#T5648-1G
Critical Commercial Assays		
Direct-zol RNA MicroPrep	Zymo Research	Cat#R2062; Lot#ZRC188234
DAB-Peroxidase IHC Kit	Abcam	Cat#ab80436
Mouse *Acta2* Taqman Probe	Applied Biosystems	Cat#Mm00725412_s1; Lot#1501761
Mouse *Beta-Actin* (Actb) Taqman Probe	Applied Biosystems	Cat#Mm00607939_s1; Lot#P151010-002
Mouse *Cxcl12* Taqman Probe	Applied Biosystems	Cat#Mm00445553_m1; Lot#1617669
Mouse *Cdkn1a* Taqman Probe	Applied Biosystems	Cat#Mm00432448_m1; Lot#1246220
Mouse *Ezh2* Taqman Probe	Applied Biosystems	Cat#Mm00468464_m1; Lot#833569
Human *Beta-Actin* (Actb) Taqman Probe	Applied Biosystems	Cat#Hs99999903_m1; Lot#P150812-005 H11
Human *Cxcl12* Taqman Probe	Applied Biosystems	Cat#H203676656_mH; Lot#P170224-001
Human *Ezh2* Taqman Probe	Applied Biosystems	Cat#H201016789_m1; Lot#P130125-005
Mouse CXCL12/SDF-1 alpha Quantikine ELISA Kit	R&D Systems	Cat#MCX120; Lot#P134406
cOmplete Mini, EDTA-free Protease Inhibitor	Roche	Cat#11836170001; Lot#13538100
Dynabeads Protein A	Invitrogen	Cat#10002D; Lot#00357670
eBioscience 1X RBC Lysis Buffer	Invitrogen	Cat#00-4333-57
Liberase TL Research Grade	Roche	Cat#5401020001
7-AAD Viability Staining Solution	BioLegend	Cat#420403
Anti-Mouse Ig, κ/Negative Control Compensation Particles Set	BD Biosciences	Cat#51-90-9001229; Lot#6236953
PolyScreen PVDF Hybridization Transfer Membrane	Perkin Elmer	Cat#NEF1002001; Lot#507409
Luminata Crescendo Western HRP Substrate	Millipore	Cat#WBLUR0100; Lot#160263
4-5% Mini-PROTEAN TGXPrecast Protein Gel	Bio-Rad	Cat#4561083
Experimental Models: Cell Lines		
293T Cells	ATCC	CRL-3216; RRID:CVCL_0063
Experimental Models: Organisms/Strains		
Mouse: C57/BL6 “Black 6”	The Jackson Laboratory	JAX: 000664; RRID:IMSR_JAX:000664
Mouse: B6(FVB)-*Cxcl12^tm1-1Link^*/J (“Cxcl12^fl/fl^”)	The Jackson Laboratory	JAX: 021773; RRID:IMSR_JAX:021773
Mouse: B6.129(Cg)-*Gt(ROSA)26Sor^tm3(ACTB-tdTomato,-EGFP)Luo^*/J (“mTmG”)	The Jackson Laboratory	JAX: 007676; RRID:IMSR_JAX:007676-UCD
Mouse: B6;129S2-*Cdkn1a^tm1Tyj^*/J (“Cdkn1a−/−“)	The Jackson Laboratory	JAX: 003263; RRID:IMSR_JAX:003236
Mouse: STOCK Tg(KRT14-cre/ERT)20Efu/J (“K14CreER+”)	The Jackson Laboratory	JAX: 005107; RRID:IMSR_JAX:005107
Mouse: K5rtTA; TetOCre	Sarah Millar	N/A
Oligonucleotides		
ChIP Mouse Cxcl12 promoter FWD: CTGTTTCGTCTCTCAGGTTCTT	Integrated DNA Technologies	N/A
ChIP Mouse Cxcl12 promoter REV: GCTGGGTCGTAGAGCTTAATG	Integrated DNA Technologies	N/A
ChIP Mouse Cxcl12 Transcription Start Site FWD: CTGTTTCGTCTCTCAGGTTCTT	Integrated DNA Technologies	N/A
ChIP Mouse Cxcl12 Transcription Start Site REV: GCTGGGTCGTAGAGCTTAATG	Integrated DNA Technologies	N/A
ChIP Mouse Cxcl12 upstream FWD: CAACATCTCCTTCTTTCCCTACC	Integrated DNA Technologies	N/A
ChIP Mouse Cxcl12 upstream REV: CTACCCTCCAACAAGCATTCA	Integrated DNA Technologies	N/A
ChIP Mouse Cxcl12 −20kb control FWD: GCCTGGAGAACCTTCTATCTTAAC	Integrated DNA Technologies	N/A
ChIP Mouse Cxcl12 −20kb control REV: GTCCACATGCAAATCTTCACAC	Integrated DNA Technologies	N/A
Recombinant DNA		
Human EZH2: 2146 CGGCTCCTCTAACCATGTTA pLKO_TRC005	The RNAi Consortium	TRCN0000039040
Software and Algorithms		
FlowJo	Treestar	RRID: SCR_008520; https://www.flowjo.com/
Other		
2mm mechanical punch	Roboz	Cat#65-9906
1.5mm biopsy punch	Acuderm	Cat#P1550
6mm biopsy punch	Delasco	Cat#DBP-6
Silicone wound splints	Grace Biolabs	SKU: 476687
Medium 154	GIBCO	Cat#M154500
Human Keratinocyte Growth Supplement	GIBCO	Cat#S0015
Keratinocyte-SFM (1×)	GIBCO	Cat#17005042
DMEM 4.5g/L glucose, L-glutamine, sodium pyruvate	Corning	Cat#10-013-CV

### CONTACT FOR REAGENT AND RESOURCE SHARING

Further information and requests for resources and reagents should be directed to and will be fulfilled by the Lead Contact, Thomas Leung (thl@pennmedicine.upenn.edu).

### EXPERIMENTAL MODEL AND SUBJECT DETAILS

#### Animals

C57BL/6 female mice (18-months old) were purchased from the National Institute on Aging, and C57BL/6 male mice (18-months old) were purchased from Jackson Labs. C57BL/6 (strain #000664, 1-month old) females, *Cxcl12^fl/fl^* (strain #021773), mTmG mice (strain #007676), *Cdkn1a*^−/−^ mice (strain #003263), and K14-CreER (strain #5107) were purchased from The Jackson Laboratory. All mice were group housed in the animal facility of University of Pennsylvania on a 12-hour light/12-hour dark cycle with *ad libitum* access to water and normal chow. *K5-rtTA; tetO-Cre* mice were a generous gift from Sarah Millar (University of Pennsylvania). Mice were verified by genotyping. To delete alleles, we administered doxycycline food pellets (6gm/kg, Bio-Serv) for one week or administered 1 mg of tamoxifen (diluted in corn oil) daily by intraperitoneal injection for 5 days. Mice used in this study were female age-matched littermates that were randomly assigned to experimental groups, except for SDF1KO^ker^ experiments where both male and female mice were used and the DZNep aged mouse experiment where male mice were used. No sex-dependent differences were observed.

#### Study approval

Experiments involving mice were reviewed and approved by the Institutional Animal Care and Use Committee of University of Pennsylvania. Mice were treated in accordance with the NIH guidelines for the humane care of animals.

#### Cell Culture and Human Skin Organoids

Primary human keratinocytes of different ages and gender were obtained from the University of Pennsylvania Skin Biology and Disease Resource Center. The specific age and location is described in Figure S4D. Cells were grown in supplemented 50:50 keratinocyte media, a 50:50 mixture of keratinocytes-SFM (Thermo Scientific) and Medium 154 (Thermo Scientific) (Leung et al., 2013). Cells are grown at 37°C and 5% CO2. For nutrient-deprivation experiments, keratinocyte media was removed and replaced with unsupplemented keratinocyte media (50:50 without supplementation) for 24-hours.

Human skin organoids were established using previously detailed methods (Ridky et al., 2010). Briefly, 1.0 × 10^6^ primary human keratinocytes were seeded onto aceulluar human dermis, in keratinocyte growth media (KGM) (DMEM high glucose, 22% Ham’s-F12, 10% Fetal Bovine Serum, 1% penicillin/streptomycin, 1% antibiotic/antimycotic, 0.2% adenine, 0.2% hydrocortisone, 0.13% insulin, 0.1% cholera toxin, 0.1% EGF, 0.1% transferrin/triiodo-L-thryonine, 0.1% ciprofloxacin hydrochloride). The organoids were maintained at the air-liquid interface at 37°C for 4 days prior to being wounded.

### METHOD DETAILS

#### Injury Models

For ear wounding, we used a standard 2mm mechanical punch (Roboz, Gaithersburg, MD) to create a hole in the center of each outer ear (pinna). Ear hole diameter was measured using a dissection microscope (Nikon) in the horizontal and vertical directions on a weekly basis. Ears were excluded if there were signs of wound infection, tearing of the ear, or abnormal geometric shape. These criteria were pre-established.

For human skin organoid injury, we used a standard 1.5mm punch (Acupunch) to create a hole in the center of each graft.

#### Murine Excisional Back Wound Model

We used a well-described murine full-thickness excisional wound model (Loh et al., 2009). Briefly, a 6mm disposable biopsy punch (Delasco) was used to make two circular full thickness wounds on the dorsal back skin of mice. Silicon wound splints (Grace BioLabs) were sutured with 4-0 Nylon to prevent skin contracture. Wounds were dressed with a sterile occlusive dressing and monitored daily. Borders were monitored by frequently application of permanent marker. Photos were taken at various time points throughout the 26-day duration of the experiment.

#### Parabiosis

Parabiosis surgery followed previously described procedures (Kamran et al., 2013). Briefly, mirror-image incisions at the left and right flanks were made through the skin. Elbow and knee joints from each parabiont were sutured together with 3-0 Nylon, and the skin of each mouse was sutured with 4-0 Nylon to the skin of the adjacent parabiont. Each mouse was treated with subcutaneous normal saline, meloxicam (Putney), and buprenorphine hydrochloride (Butler Schein Animal Health) as directed for pain and monitored closely during recovery. 2-3 days after surgery, steristrips were placed over the sutures on the skin. For overall health, several recovery characteristics were analyzed at various times after surgery, including weight and grooming responses, and animals were excluded if they failed overall health inspection. We waited 1 month after the parabiosis surgery to perform our standard ear punch assay.

#### Histology and Immunohistochemistry

Standard histology and immunostaining protocols were performed, and investigators were blinded during histologic staining. Briefly, immunohistochemical analysis was performed on 5-10 μm-thick sections of mouse skin. The following primary antibodies were used: mouse monoclonal anti-Ki-67 (1:100, Immunostar, 1:100, Abcam, ab15580), rabbit polyclonal anti-SDF1 (1:100, Abcam, ab25117), rabbit monoclonal anti-EZH2 (1:100, Cell Signaling Technology, #5246), rabbit polyclonal anti-αSMA (1:100, Abcam ab7817), rabbit polyclonal anti-keratin 14 (1:100; Bio Legend), rabbit polyclonal anti-keratin 10 (1:100; Bio Legend), and rabbit polyclonal anti-loricrin (1:100; Bio Legend). Immune complexes were detected with secondary antibodies conjugated with either GFP or DAB-Peroxidase substrate solution (Abcam). Trichome staining was performed per the manufacturers directions (Thermo Scientific). After staining, images were directly analyzed on an AxioM1 microscope equipped with a CCD digital camera (Carl Zeiss) or Keyence imaging system. A minimum of 4-6 sections was stained per sample. Secondary only control was included for every experiment. Un wounded skin was included as a control for each antibody. Representative images selected for figure panels.

Relative protein expression was quantified on a 0 to 3 scale: 0 indicates no staining; 1 signifies weak staining; 2 signifies intermediate staining; and 3 signifies strong staining. For EZH2, a score was obtained every 250 μm of epidermis (minimum of 1mm) for each sample and then averaged. Our prior work with cartilage-lineage tracing regenerating mice (Cdkn1a^−/−^, Col2-CreER, mTmG,(Leung et al., 2015)) agrees with other published studies that ear chondrocyte regeneration occurs as individual islands at the distal tip (Zhang et al., 2015). For Ki-67+ chondrocytes, positive cells were counted for every 250-400 μm of cartilage, beginning at the distal tip for each sample. As an internal positive control, we counted Ki-67+ basal keratinocytes. For quantification of SDF1 and αSMA staining, we used open-source software to count colored pixels as previously described (Billings et al., 2015). A minimum of 4 different samples were used for each time point and then averaged.

#### Real-time RT–PCR

Freshly dissected tissue from the rim of healing appendages were manually dissociated with scissors, collected in TRI Regeant (Zymo) and then mechanically disrupted (Fastprep 24, Lysing Matrix D, MP Bio). Total RNA was isolated by Direct-Zol RNA MicroPrep (Zymo). RNA concentration was measured by Nanodrop 1000 (Thermo Scientific). cDNA synthesis was performed with Maxima Reverse Transcriptase (Thermo Scientific) per manufacturer’s instructions. One-step quantitative RT–PCR was performed and analyzed using an ABI ViiA7 Real-Time PCR detection system (Applied Biosystems) with TaqMan one-step RT–PCR Master Mix Reagents. Taqman probes were purchased for mouse *(Acta2, beta-actin, Cxcl12, Cdkn1a, Ezh2*) and human genes (*beta-actin, Cxcl12, Ezh2*) (Applied Biosystems).

#### Chromatin Immunoprecipitation (ChIP)

Briefly, dissected ear tissue was treated with 4% formaldehyde solution and quenched with glycine. Tissue was frozen in liquid nitrogen and pulverized (Covaris). Tissue was lysed in ChIP lysis SDS buffer (50mM HEPES/NaOH pH 7.5, 1% SDS, 10mm EDTA) supplemented with protease and phosphatase inhibitors (Roche). ChIP buffer (50mM HEPES/NaOH pH 7.5,140mM NaCl, 1% Triton X-100, 0.1% Na-Deoxycholate, 1mmEDTA, 0.1% SDS) was added to the samples, and sonication was performed (Covaris). After centrifugation, antibody (H3K27me3 (Cell Signaling Technologies, #9733), H3K4me3 (Millipore, #07-473), EZH2 (Millipore, Clone AC22, #17-662)) was added to each sample and samples rotated overnight at 4°C. Protein-A/G magnetic beads (Dynabeads; Invitrogen) were washed and added to samples for 4 hours. Beads were collected, washed thoroughly with ChIP wash buffer (50mM Tris pH8.0, 250mM LiCl, 0.5% NP-40, 0.5% Na-deoxycholate, 1mM EDTA), and resuspended in decrosslinking buffer (50mM Tris pH 8.0, 10mM EDTA, 1% SDS). After RNase A and proteinase K treatment, samples underwent phenol-chloroform extraction and ethanol precipitation. Samples were analyzed by real-time PCR. ChIP primers available in Key Resources Table.

#### Flow Cytometry and Cell Sorting

To assess GFP+ cells in parabiosis mice, blood was obtained from the retro-orbital sinus, and red blood cells were removed (RBC lysis buffer, eBioscience). Cells were resuspended in FACS buffer (HBSS, 0.2% BSA, 1% HEPES). For keratinocyte isolation experiments, freshly dissected tissue from the rim of healing wounds were manually dissociated with scissors and then incubated with Liberase TL (Roche) for 90 min at 37°C. After dissociation, cells were washed in PBS and resuspended in FACS buffer (PBS + 0.05% NaN3 + 2% FBS). 7-AAD (BioLegend) was added to exclude dead cells. GFP+ cells were identified and sorted.

Cell sorting was performed at the Flow Cytometry and Cell Sorting Facility (University of Pennsylvania). Standard FACS procedures were performed. The BD CompBeads Set Anti-Mouse Ig, κ was used to compensate and determine gating strategies. GFP expression was quantified with FlowJo software (Treestar). Sorted cells were immediately harvested for RNA purification.

#### ELISA

Blood was obtained from the retro-orbital sinus, and serum was collected after blood coagulation. Mouse SDF1 ELISA was performed per the manufacturer’s instructions (R&D Systems, #MCX120). Recombinant SDF1 was used as a positive control.

#### Knockdown Experiments

We obtained seven lentiviral knockdown vectors from The RNAi Consortium shRNA Library for human EZH2 (TRCN0000039040). Control knockdown vector is a lentivirus vector containing a scrambled sequence. Lentivirus was produced as previously described (Leung et al., 2004) and used to infect primary keratinocytes. After antibiotic selection, knockdown vectors were assessed for efficacy by Western analysis. The top 2 vectors (#62 and #28) were used to create EZH2-knockdown keratinocytes, and the data shown is from #28 knockdown vector-infected keratinocytes. Cells were then used for nutrient-deprivation experiments as described above.

Total protein was prepared from mouse ear tissue by mechanical tissue disruption in PBS with protease inhibitor and an equal volume of 2× SDS sample buffer was then added. Equal amounts of protein were resolved on SDS-PAGE (Bio-Rad) and transferred to PVDF membranes (Perkin Elmer) for immune blotting with specific antibodies, including rabbit monoclonal anti-EZH2 (1:1000; Cell Signaling Technology, 5246) and rabbit polyclonal anti-β-actin-HRP (1:1000; Santa Cruz Biotechnology). After further incubation with HRP-conjugated secondary antibodies, signal was visualized using ECL detection (Millipore Luminata) on film.

#### Pharmacologic inhibitor experiments

Mice treated with DZNep received IP injections (2 mg/kg, Millipore #252790) three-times per week; mice were pre-treated for one week before receiving ear punch.

Organoids treated with DZNep (1μM final concentration, Millipore #252790) received the inhibitor one day prior to injury and continued until sample collection.

#### Primary human skin microarray

Data was accessed on the Gene Expression Omnibus website (GEO Accession numbers: GSE84571, GSE28914, GSE33169). Uninjured and 7-day post-injured human skin samples of different ages were identified, and levels of Cxcl12 (ID: 203666_at) was assessed directly on the GEO website.

### QUANTIFICATION AND STATISTICAL ANALYSIS

#### Experimental Design

Presented data combines all experiments, and unless noted, all experiments were repeated 3 times independently. Experiments were not randomized, and investigators were not blinded to allocation during experiments and outcome assessment, unless noted in the text.

#### Statistical Analysis

For *in vivo* time courses comparing hole size (Figures 1B, 2B, 3G, 4F, S2C, S3B), data were analyzed using the method of Generalized Estimating Equations (GEE). The temporal correlation and correlation between holes within the same mouse were modeled using an exchangeable correlation matrix. The GEE model incorporated a temporal main effect, a main effect comparing the types of mice, and an interaction of the two main effects. Statistical significances in the GEE model were determined using a Wald test.

For parabiosis experiments, a pre-hoc power analysis was performed using “long power” on R. We tried three different correlation values (0.2, 0.5, 0.8) and two values of variance in the areas (0.25, 0.5) to detect a difference of d = 0.5mm between the two groups. The sample size needed ranged from 1-6 pairs for a power of 80% and 2-sided-significance of 5%. A post hoc power analysis was also performed using power calculator on R. Given our mean difference in surface area and the sample size of n = 3, we achieved greater than 95% power detection.

Otherwise, 2-tailed Student’s t test was used to determine significance, with *P values* of less than 0.05 considered significant. Higher levels of significance are indicated by the following: ** p < 0.01, *** p < 0.001 in the text. When appropriate, specific P values are provided in figure legends.

## Supplementary Material

1

## Highlights

- Full-thickness skin wounds in aged but not young mice fully regenerate
- Genetic deletion of SDF1 in young skin enhanced tissue regeneration
- Aging remodels chromatin accessibility at the SDF1 gene to inhibit SDF1 transcription
- Human skin also exhibits age-dependent SDF1 suppression
