# Aging Suppresses Skin-Derived Circulating SDF1 to Promote Full-Thickness Tissue Regeneration

**DOI:** 10.1016/j.celrep.2018.12.056

**Published:** 2018-12-26

**Authors:** Mailyn A. Nishiguchi, Casey A. Spencer, Denis H. Leung, Thomas H. Leung

In the originally published version of this article, the *Acomys* (spiny mouse) is incorrectly referred to as a hamster strain. It is a member of the rodent family (Muridae). Also, the reference cited for *Acomys* regeneration was inadvertently modified during publication. Seifert et al. (2012a) was incorrectly referenced as Seifert et al. (2012b). Finally, a reference for the discussion on partial versus complete ear closure was accidentally omitted (Costa et al., 2009). The main text and references have now been corrected online.

The authors regret these errors.

