# Supplementary material for: Aging Suppresses Skin-Derived Circulating SDF1 to Promote Full-Thickness Tissue Regeneration
Source: Cell Rep. Author manuscript; Available in PMC 2018 Nov 28. (PMC6261459; doi:10.1016/j.celrep.2018.08.054)
Supplement: 1 [file NIHMS1508940-supplement-1.pdf]

Cell Reports, Volume *24*

Supplemental Information

Aging Suppresses Skin-Derived Circulating SDF1 to Promote Full-Thickness Tissue Regeneration

Mailyn A. Nishiguchi, Casey A. Spencer, Denis H. Leung, and Thomas H. Leung

Aging suppresses skin-derived circulating SDF1

to promote full-thickness tissue regeneration

Authors: Mailyn A. Nishiguchi[1,4], Casey A. Spencer[1,4], Denis H. Leung[3], Thomas H. Leung[1,2,5*]

Comprehensive Supplemental Information

Figure S1-S4

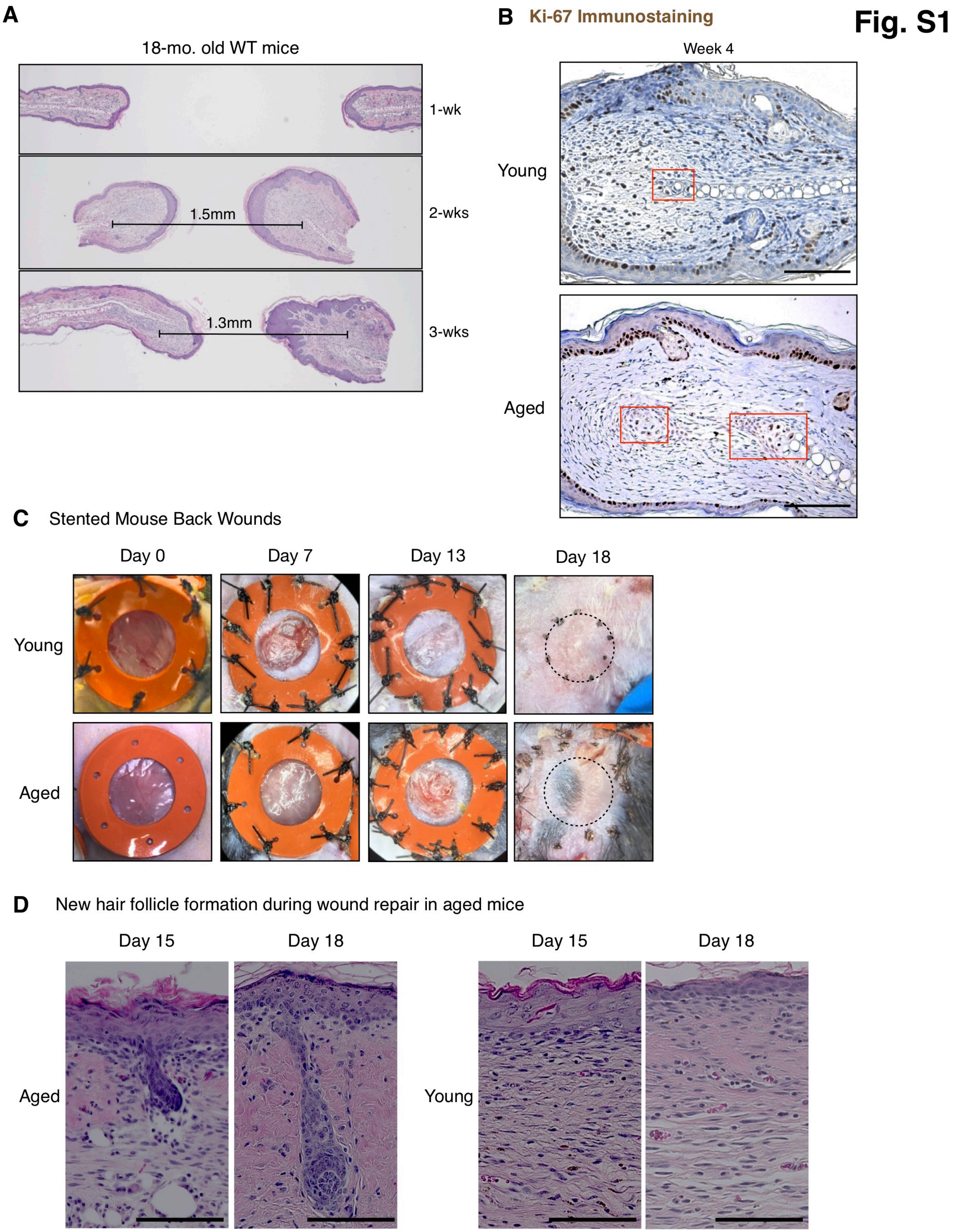

Fig. S1
A
18-mo. old WT mice
1-wk
2-wks
3-wks
1.5mm
1.3mm
B
Ki-67 Immunostaining
Week 4
Young
Aged
C
Stented Mouse Back Wounds
Day 0
Day 7
Day 13
Day 18
Young
Aged
D
New hair follicle formation during wound repair in aged mice
Day 15
Day 18
Day 15
Day 18
Aged
Young

Supplemental Information

Figure S1, Related to Figure 1: **Aging promotes tissue regeneration and diminishes scar formation in mice skin.**

(A) H&E staining of wound edge tissue taken from aged WT mice at weeks 1-3. *N=20.* Solid vertical lines indicate the ends of the cartilage plate.

(B) Ear tissue from injured aged mice demonstrate increased chondrocyte proliferation. Ki-67 immunostaining (brown cells). Red squares identify areas of Ki-67 positive chondrocytes. *N=4*.

(C) Stented mouse back wounds: representative photographs of young and aged WT mice at different time points. *N=5*.

(D) New hair follicle formation during dorsal back skin repair in aged mice. Representative H&E staining of wound edge tissue taken from young and aged WT mice at Day 15 and Day 18 after injury. *N=3.* Scale bars, 100μM.

Fig. S2

A Parabiosis Chimerism

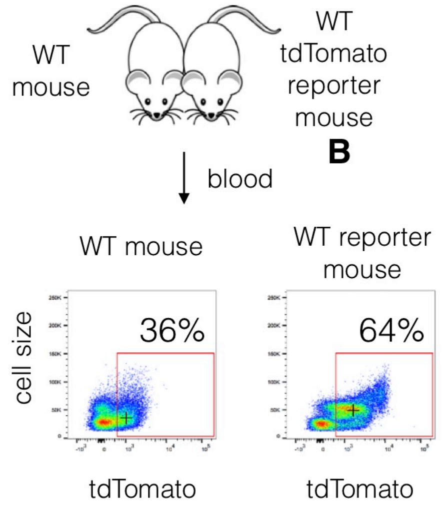

B

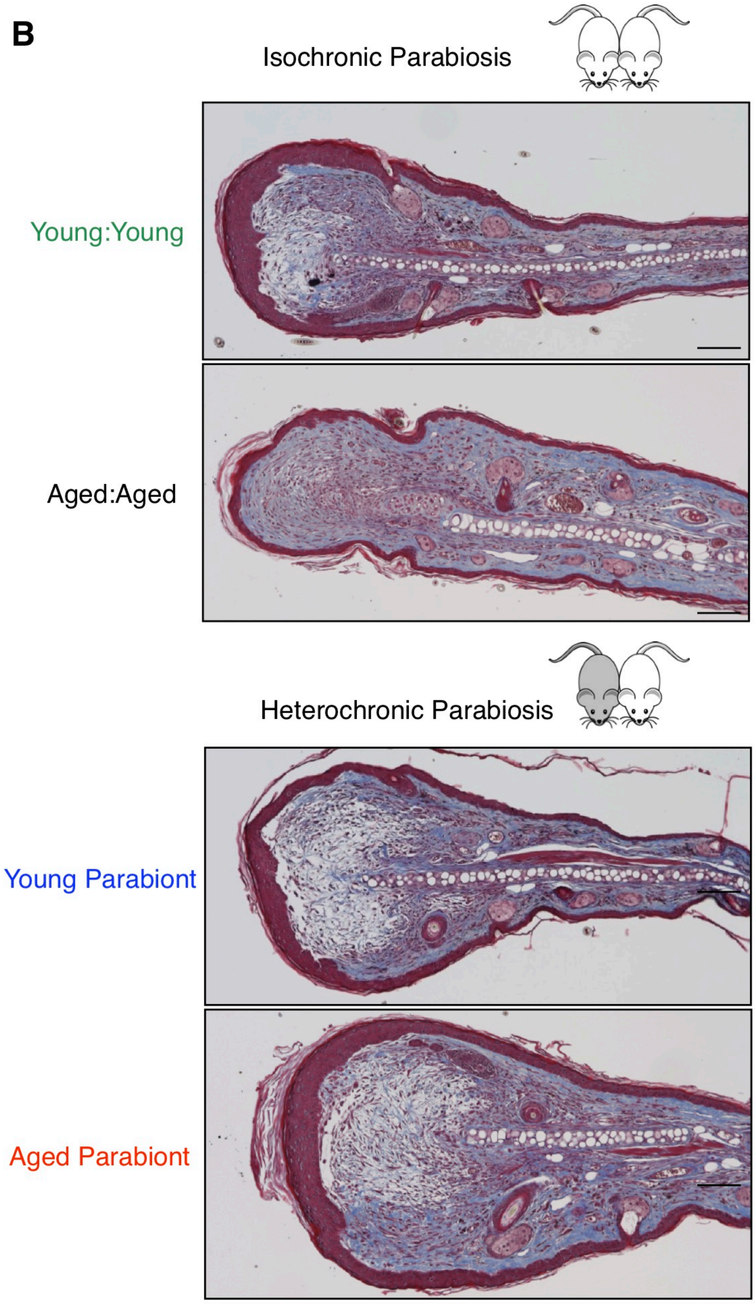

C Extending Time Between Parabiosis and Injury

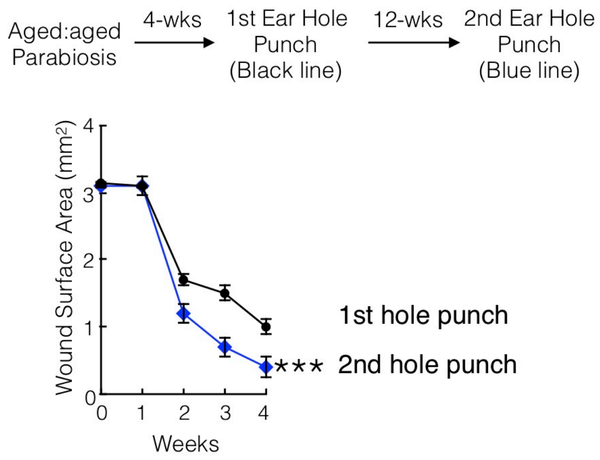

D FACS Gating Scheme for Isolating Keratinocytes and Dermal-based cells

WT K14-CreER^{mTmG}

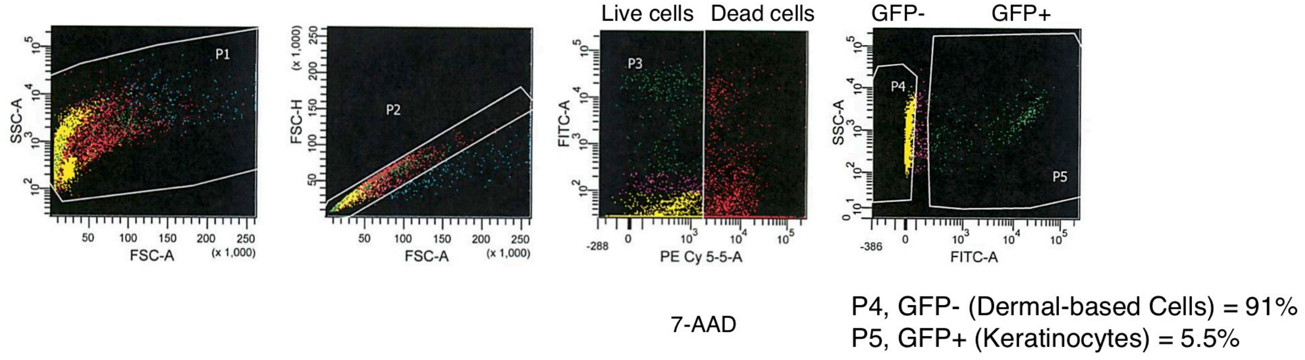

Figure S2, Related to Figure 2: Aged parabionts from heterochronic parabiosis pairs adopt the young parabiont phenotype.

(A) Parabiosis chimerisim: Representative FACS plot of blood taken from a parabiosis pair consisting of a fluorescent reporter mouse (R26R^{mTmG}, Jackson Labs) and age-matched control mouse (C57/BL6, Jackson Labs). *N=5*. WT mouse blood percentage of Td-Tomato+ cells ranged between 36-45%, which is similar to previously published studies (Conboy et al., 2005). Experiment repeated 3 times independently.

(B) Trichrome staining of ear wound-edge tissue from parabiosis pairs. Low magnification images to demonstrate consistent background Trichrome staining. *N=5.*

(C) Extending the time period between parabiosis procedure and ear injury improves ear hole closure in aged:aged isochronic pairs. After 12-weeks, a second distinct ear hole punch was performed and followed. *N=3*. (***) P=1.5e-13.

(D) Representative FACS gating scheme for keratinocyte and dermal-based cell isolation from WT K14-CreER^{mTmG} mice.

N=biological replicates per group. Error bars are s.e.m.

Fig. S3

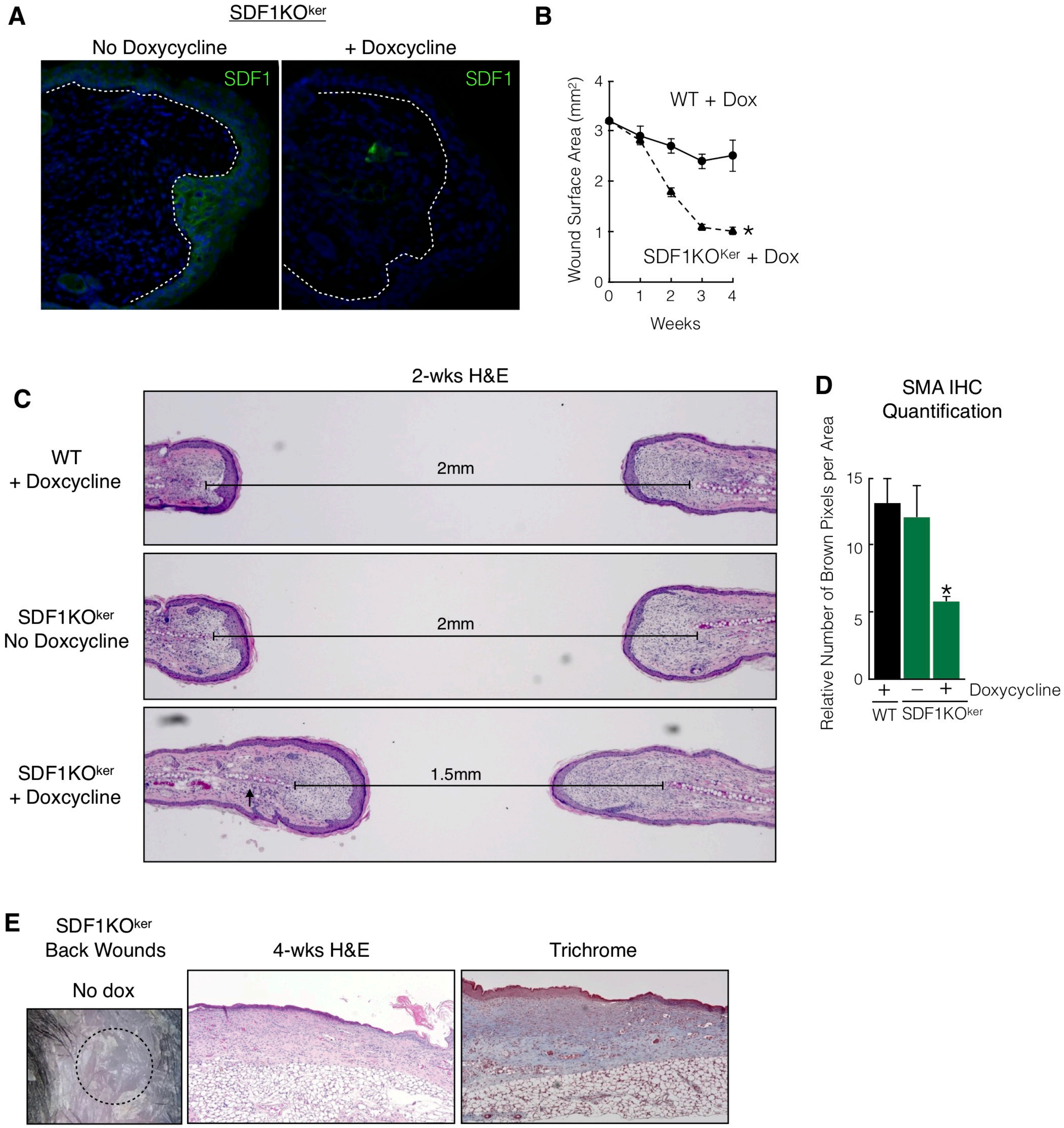

A
SDF1KOker
No Doxycycline
+ Doxcycline
SDF1
SDF1
B
Wound Surface Area (mm2)
WT + Dox
SDF1KOKer + Dox
Weeks
0
1
2
3
4
*
C
2-wks H&E
WT
+ Doxcycline
2mm
SDF1KOker
No Doxcycline
2mm
SDF1KOker
+ Doxcycline
1.5mm
D
SMA IHC
Quantification
Relative Number of Brown Pixels per Area
15
10
5
0
*
+
–
+
Doxycycline
WT
SDF1KOker
E
SDF1KOker
Back Wounds
No dox
4-wks H&E
Trichrome

Figure S3, Related to Figure 3: Young skin-specific SDF1 knockout mice ($SDF1KO^{ker}$) exhibit improved ear hole closure.

(A) Doxycycline treatment specifically deletes SDF1 expression in the skin of $SDF1KO^{ker}$ mice. SDF1 immunostaining (green) of wound edge tissue taken from control (no doxycycline treatment) or doxycycline-treated $SDF1KO^{ker}$ mice at 1-week post-injury. Dotted line identifies epidermal-dermal border. *N=6.*

(B) Doxycycline treatment does not induce ear hole closure in young WT mice. Ear hole measurements from doxycycline-treated $SDF1KO^{ker}$ and doxycycline-treated WT littermate control mice on weeks 0-4. *N=5.* (*) P=0.01.

(C) Doxycycline-treated $SDF1KO^{ker}$ mice exhibit improved chondrocyte proliferation and absence of scar formation. H&E staining of wound edge tissue taken from control (WT mice treated with doxycycline and $SDF1KO^{ker}$ mice without doxycycline treatment) and doxycycline-treated $SDF1KO^{ker}$ mice at 2-weeks post-injury. Solid vertical lines indicate the ends of the cartilage plate. Black arrow indicates cartilage regeneration. *N=6.*

(D) Doxycycline-treated $SDF1KO^{ker}$ mice exhibit less αSMA expression 2-weeks after ear injury. Quantification of αSMA immunostaining (Fig. 3E). *N=6.* (*) P=0.04

(E) Non-doxycycline-treated $SDF1KO^{ker}$ back wounds (control) heal with a fibrotic scar: photographs, H&E staining, and Trichrome staining at 4-weeks post-injury. *N=5.*

N=biological replicates per group. Error bars are s.e.m. Scale bars, 100µM.

Fig. S4

A

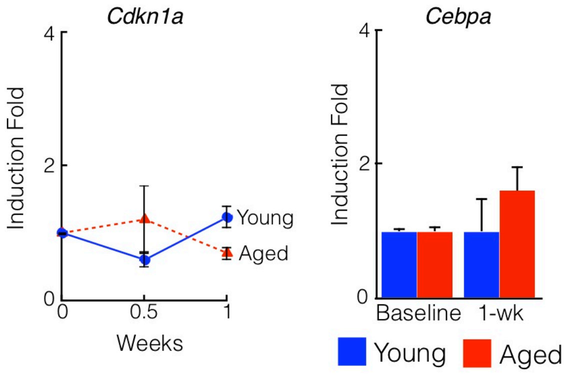

B

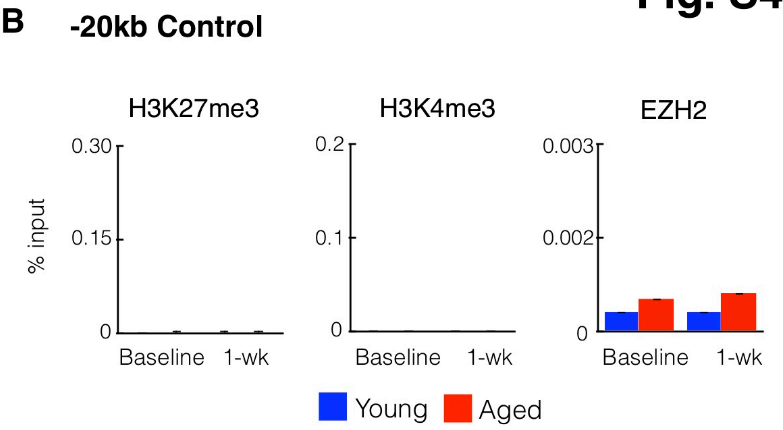

C

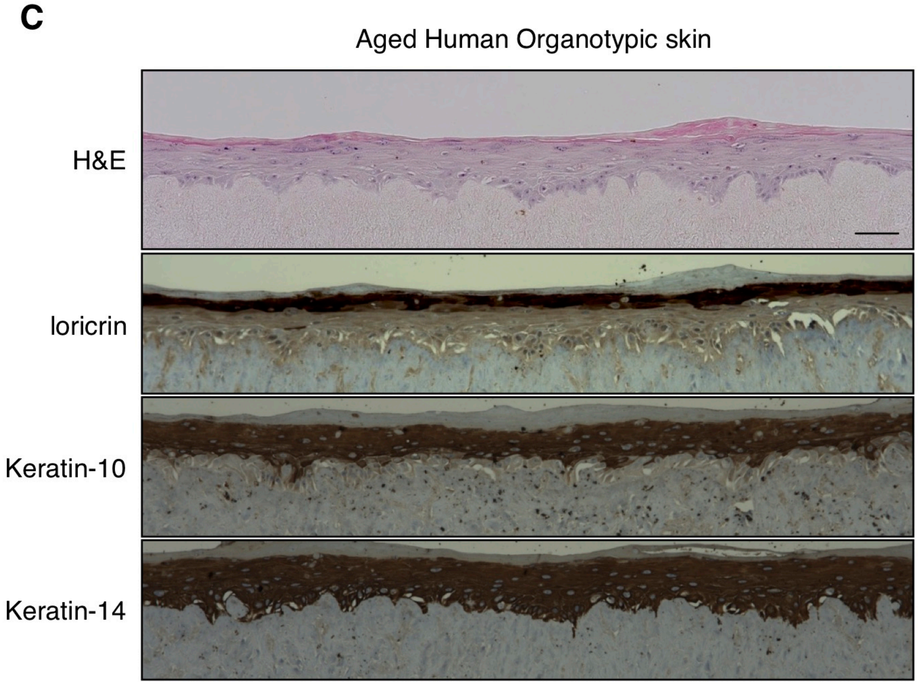

D

Primary Human Keratinocytes used for *in vitro* experiments

**Age (years)**	**Location**	**Passage**
<1	Foreskin	4
64	Face	6
72	Forearm	8
80	Scalp	5
99	Scalp	5

Figure S4, Related to Figure 4: Mouse and human skin exhibit age-dependent EZH2-mediated SDF1 induction.

(A) Known transcriptional regulators of SDF1 are unchanged with age. Relative mRNA levels of *Cdkn1a* and *Cebpa* in wound edge tissue taken from young or aged WT mice at baseline and 1-week post-injury. *N=6* mice per time point.

(B) Absence of H3K27me3, H3K4me3, and EZH2 enrichment at a control region located 20kb away from the SDF1 gene. *N=6* mice per group.

(C) Aged human organoids exhibit appropriate skin differentiation program. H&E staining (upper panel) and loricrin, keratin-10, and keratin-14 immunostaining (lower panels, brown) of aged human skin organoid. *N=5*.

(D) Table describing source of primary human keratinocytes, including: age, location, and passage number.

N=biological replicates per group. Error bars are s.e.m. Scale bars, 100μM.
